# Machine Learning Approaches for Stroke Risk Prediction: Findings from the Suita Study

**DOI:** 10.3390/jcdd11070207

**Published:** 2024-07-01

**Authors:** Thien Vu, Yoshihiro Kokubo, Mai Inoue, Masaki Yamamoto, Attayeb Mohsen, Agustin Martin-Morales, Takao Inoué, Research Dawadi, Michihiro Araki

**Affiliations:** 1 Artificial Intelligence Center for Health and Biomedical Research, National Institutes of Biomedical Innovation, Health and Nutrition, 3-17 Senrioka-Shinmachi, Settsu 566-0002, Japan; ngnmai1412@gmail.com (M.I.); m.yamamoto@nibiohn.go.jp (M.Y.); attayeb@nibiohn.go.jp (A.M.); agusmartinmorales@gmail.com (A.M.-M.); dawadi-research@nibiohn.go.jp (R.D.); 2 National Cerebral and Cardiovascular Center, 6-1 Kishibe-Shinmachi, Suita 564-8565, Japan; ykokubo@ncvc.go.jp; 3 Department of Cardiac Surgery, Cardiovascular Center, Cho Ray Hospital, Ho Chi Minh City 72713, Vietnam; 4 Faculty of Informatics, Yamato University, 2-5-1 Katayama, Suita 564-0082, Japan; takaoapple@gmail.com; 5 Graduate School of Medicine, Kyoto University, 54 Shogoin-Kawahara-cho, Sakyo-ku, Kyoto 606-8507, Japan; 6 Graduate School of Science Technology and Innovation, Kobe University, 1-1 Rokkodai Nada-ku, Kobe 657-8501, Japan

**Keywords:** stroke, supervised machine learning, unsupervised machine learning, logistic regression, random forest, support vector machine (SVM), extreme gradient boost (XGBoost), light gradient boosted machine (LightGBM), k-prototype clustering, Shapley Additive Explanations (SHAP)

## Abstract

Stroke constitutes a significant public health concern due to its impact on mortality and morbidity. This study investigates the utility of machine learning algorithms in predicting stroke and identifying key risk factors using data from the Suita study, comprising 7389 participants and 53 variables. Initially, unsupervised k-prototype clustering categorized participants into risk clusters, while five supervised models including Logistic Regression (LR), Random Forest (RF), Support Vector Machine (SVM), Extreme Gradient Boosting (XGBoost), and Light Gradient Boosted Machine (LightGBM) were employed to predict stroke outcomes. Stroke incidence disparities among identified risk clusters using the unsupervised k-prototype clustering method are substantial, according to the findings. Supervised learning, particularly RF, was a preferable option because of the higher levels of performance metrics. The Shapley Additive Explanations (SHAP) method identified age, systolic blood pressure, hypertension, estimated glomerular filtration rate, metabolic syndrome, and blood glucose level as key predictors of stroke, aligning with findings from the unsupervised clustering approach in high-risk groups. Additionally, previously unidentified risk factors such as elbow joint thickness, fructosamine, hemoglobin, and calcium level demonstrate potential for stroke prediction. In conclusion, machine learning facilitated accurate stroke risk predictions and highlighted potential biomarkers, offering a data-driven framework for risk assessment and biomarker discovery.

## 1. Introduction

Stroke is a major global health concern, with high rates of disability and mortality worldwide. In 2019, stroke was responsible for 11% of the 55.4 million deaths worldwide [1]. According to the Global Stroke Factsheet published in 2022, the risk of stroke has increased by 50% in the last 17 years [2]. Stroke incidence, mortality, prevalence, and disability-adjusted life years (DALY) have also increased significantly over the past few decades [2]. The economic burden of stroke is also substantial, with global direct and indirect costs totaling USD 891 billion in 2017 [3].

Early detection of stroke risk by predictive models can enable early intervention and prevention, reducing the severity of stroke events. Predictive models can improve stroke patient outcomes, including reduced morbidity and mortality, and enable personalized therapy. Furthermore, stroke prediction can inform resource allocation and prioritization of preventive measures in communities where they are most needed. Prediction models can also provide insights into the underlying causes of stroke, leading to the development of new treatments and preventative measures. Thus, research on the prediction of stroke and the identification of high-risk populations is crucial.

Population-based cohort studies have been used to identify stroke risk, which follows a specific population over time, and collect data on various factors such as demographics, medical history, lifestyle habits, and disease outcomes. However, conventional risk scores have limitations due to the complex interactions among diverse factors in real-life situations. Machine learning (ML) algorithms can provide a technical solution to these difficulties by automatically selecting the most important features and variables, reducing the need for manual feature selection. Additionally, ML algorithms can be trained on large datasets and typically achieve greater accuracy than traditional statistical methods, especially for complex interactions between variables, making them more generalizable to new and unexplored data.

While ML techniques have been used in increasing cardiovascular event-related studies in recent years, they remain a relatively unexplored topic in stroke research [4,5,6,7]. Therefore, combining unsupervised and supervised ML techniques to identify high-risk groups is essential for stroke prediction. Additionally, the SHAP approach can be utilized to determine the importance of independent variables with stroke and explore the potential of unidentified risk factors for stroke prediction.

In summary, this study aims to explore the role of machine learning techniques in predicting stroke incidence and uncovering novel risk factors. Through a comprehensive ML-driven analysis, we endeavor to enhance our understanding of stroke risk assessment and contribute to the development of effective preventive strategies.

## 2. Materials and Methods

### 2.1. Study Participants

The Suita Study, a prospective population-based cohort study, was conducted in Suita city, located in northern Osaka, Japan. The study design and selection criteria have been previously described [8,9]. Between 1989 and 1999, the study enrolled 7672 men and women aged 30 to 84 years who did not have clinical cardiovascular disease at baseline. The participants were selected from the municipality population registry and followed for an average of 15 years until their first stroke, myocardial infarction (MI), death, or relocation. All participants who agreed to participate in the Suita study provided informed consent.

Data were prospectively collected, including demographics, medical history, medical imaging and laboratory data, lifestyle habits, and outcome. Data collection is described elsewhere. These evaluations are the baseline examination for the present investigation [8,9].

### 2.2. Outcomes

In this study, stroke was rigorously defined according to the U.S. National Survey of stroke criteria. Strokes were classified into subtypes based on imaging and autopsy findings, including cerebral infarction (thrombotic or embolic), intracerebral hemorrhage, and subarachnoid hemorrhage. CT scans were used as the primary imaging modality to confirm the diagnosis of stroke and to classify its subtype. MRI was employed for further detailed assessment, especially in cases where CT results were inconclusive or additional information was required to differentiate between stroke subtypes. In cases where patients had undergone autopsy, the findings were used to corroborate the stroke diagnosis and classification.

### 2.3. Risk Factors and Additional Measurements

The baseline measurements were collected before the occurrence of stroke events. The participant’s blood pressure (BP) was measured through a standardized protocol for accuracy and precision, utilizing a mercury column sphygmomanometer and a suitable cuff. Participants were instructed to rest for at least 5 min before their initial BP measurement to establish a stable baseline. To ensure proper observation and recording, two separate BP readings were taken at intervals greater than one minute and averaged. Hypertension was defined as systolic blood pressure ≥ 140 mmHg, diastolic blood pressure ≥ 90 mmHg, or using antihypertensive medications. Body mass index (BMI) was calculated as weight (kg) divided by the square of height (m^2^). As part of the baseline evaluation, routine blood tests were performed, measuring serum total cholesterol and high-density lipoprotein cholesterol, as well as glucose levels. Non-HDL cholesterol was calculated by total cholesterol concentration minus high-density lipoprotein cholesterol. Diabetes was defined as fasting plasma glucose ≥ 126 mg/dL, and/or using diabetic medications. Metabolic syndrome was defined as a combination of abdominal obesity, impaired fasting glucose, atherogenic dyslipidemia, and elevated blood pressure. The original Japanese criteria for metabolic syndrome were the presence of high waist circumference ≥ 85 cm in men and ≥90 cm in women and/or BMI ≥ 25.0 kg/m^2^, an essential component plus ≥ 2 (definite MetS) of the followings [10,11]: (1) systolic blood pressure ≥ 130 mm Hg and/or diastolic blood pressure ≥ 85 mm Hg or medication use; (2) triglyceride level ≥ 150 mg/dL and/or HDL cholesterol level < 40 mg/dL; and (3) fasting glucose level ≥ 100 mg/dL and/or medication use. Estimated glomerular filtration rate (eGFR) (mL/min/1.73 m^2^) was calculated according to the original Modification of Diet in Renal Disease (MDRD) equation modified by the Japanese coefficient (0.881) as follows [12]:

$$\mathrm{eGFR}=0.881\times 186\times {\left\{\mathrm{serum}\;\mathrm{creatinine}\right\}}^{-1.154}\times {\left\{\mathrm{age}\right\}}^{-0.203}\times \left(0.742\;\mathrm{for}\;\mathrm{female}\right).$$

### 2.4. Statistical Methods

Data are presented as percentages, means (standard deviations), or medians (IQRs) depending on variable characteristics. Chi-squared tests or Fisher exact tests were used for categorical variables, whereas t-tests, analyses of variance, or Kruskal–Wallis tests were used for continuous variables.

**Figure 1:**
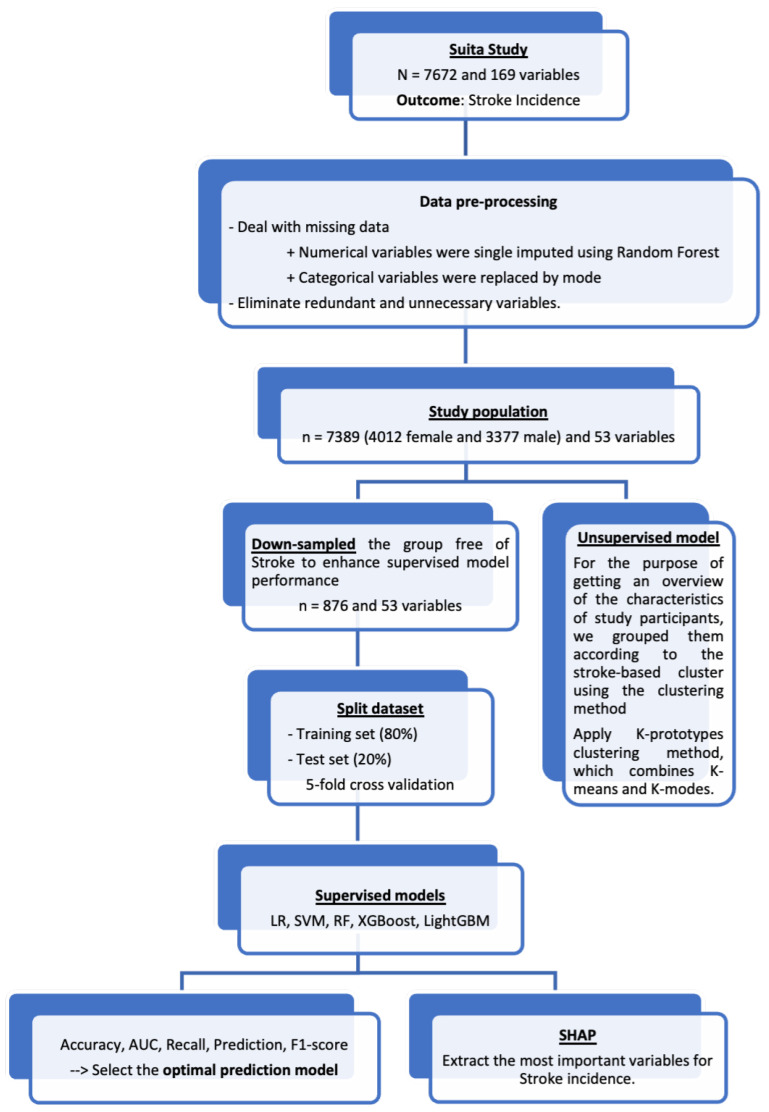
A flow chart visualizing the model development process.

The flowchart, in Figure 1, represents the development of a stroke prediction model.

### 2.5. Data Pre-Processing

Our original dataset consisted of 7672 participants and 169 variables. We utilized a random forest method to impute missing values for continuous variables. For missing categorical variables, we replaced them with the mode before applying one-hot encoding.

In order to facilitate the interpretation of results, it is necessary for all variables to be expressed in the same unit. Therefore, we normalize all these variables using standardization methods (or Z-score normalization) that involves centering the variable mean at 0 and standardizing the variance at 1 [13]. The procedure involves subtracting each observation’s mean and dividing by the standard deviation.

We extracted all variables from the raw dataset to build a data-driven model. We removed multicollinearity by considering the clinical meaning of variables, variance inflation factor (VIF), and correlation coefficients between variables. After that, we have to eliminate the redundant and unneeded variables from the dataset, which included 7389 participants (4012 female and 3377 male) and 53 variables.

### 2.6. Unsupervised Learning

For the purpose of obtaining an overview of the characteristics of study participants and their risk factors in the association with stroke incidence, we used the clustering method that is a type of unsupervised learning that consists of similar characteristics within a group and different characteristics between groups through the characteristics of individuals. We employed the k-prototypes clustering approach, combining the k-means and k-modes clustering methods because our dataset had continuous and categorical variables [14].

Initially, we determined the optimal number of clusters by employing techniques such as Elbow optimization and Silhouette scoring. The unsupervised k-prototype approach effectively identified three distinct clusters in the dataset, which correspond to certain risk groups.

### 2.7. Supervised Learning

In this study, we took several steps to ensure the robustness of our supervised models for predicting stroke incidence. Initially, we addressed the challenge of imbalanced data by down-sampling the group free of stroke while maintaining the original ratio of positive (stroke) to negative (free of stroke) samples. Subsequently, we randomly divided the dataset into training (80%) and testing (20%) sets.

Following this, we trained five supervised models using the training set, employing logistic regression (LR), random forest (RF), Support Vector Machine (SVM), Extreme Gradient Boost (XGBoost), and Light Gradient Boosting Machine (LightGBM). We employed a combination of 5-fold cross-validation. The cross-validation process involved iteratively training the models on different subsets of the training data and validating them on the remaining subsets to ensure robustness and prevent overfitting.

For hyperparameter tuning, we utilized the Optuna framework (version 3.0.4), which is an efficient and flexible hyperparameter optimization method [15]. Optuna employs a sophisticated search algorithm that automates the process of hyperparameter tuning by exploring a wide range of hyperparameter values for each model.

Each model has unique strengths that could make it the best model for predicting stroke in our cohort. LR is a simple approach to prediction that provides baseline accuracy scores for comparisons with other non-parametric machine learning models [16]. RF is a bagging technique that generates many decision trees to reduce the high variance of decision trees [15]. SVM classifies data by separating classes with a boundary [17,18], while XGBoost is a powerful tool for classification and regression [18]. LightGBM is a fast, distributed, high-performance gradient-boosting framework that uses a tree-based learning algorithm [19].

For evaluating the efficacy of these models, we calculated using several performance metrics, such as accuracy, area under the receiver operating characteristic curve (AUC), recall, precision, and F1-score, using the test set. The metrics’ explanation is included in Appendix A, Table A1.

### 2.8. Extraction of Important Variables for Stroke Risk

To determine which predictors contribute the most to the prediction model, we applied the Shapley Additive Explanations (SHAP) method [13]. SHAP is a unified framework for predictive interpretation that expresses variables’ importance by comparing situations’ predictions with baseline values when there are specific values for a given feature [13,18].

## 3. Results

In total, 7389 individuals participated in our Suita study. At baseline, the median (interquartile range) age was 56 (44–65) years, 45.7% of participants were male, and 5.9% had suffered a stroke. People with incident stroke were older and more likely to have a higher prevalence of hypertension, diabetes, and metabolic syndrome. As demonstrated in Table 1, the incidence of stroke was higher among men and those with a higher body mass index, blood sugar, triglyceride, and non-high-density lipoprotein cholesterol level but a low estimated glomerular filtration rate (eGFR).

**Figure 2:**
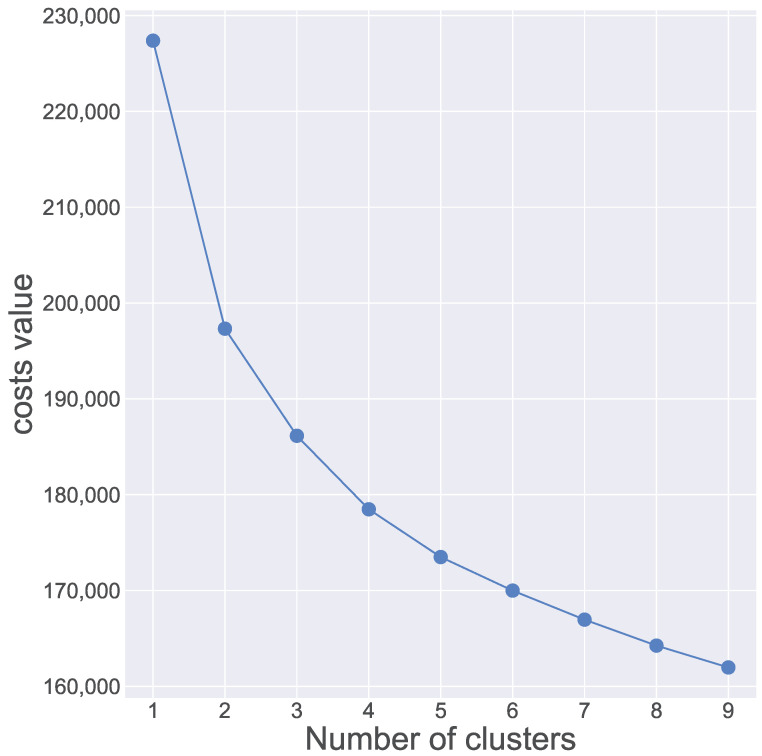
Elbow plot for determining optimal number of clusters. The elbow method for determining the optimal number of clusters in clustering algorithms like k-means involves plotting the Within-Cluster Sum of Squares (WCSS) against the number of clusters (k), and identifying the “elbow point” where adding more clusters does not significantly reduce the WCSS. The elbow point seems to be around k = 3 or k = 4, where the WCSS starts to decrease more slowly.

**Figure 3:**
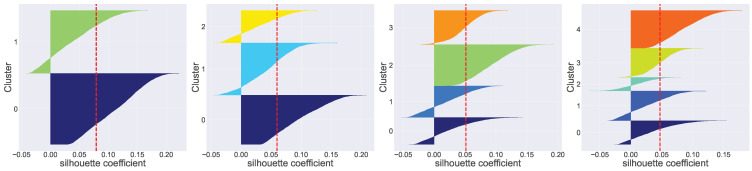
Silhouette plots (with k from 2 (**left**) to 5 (**right**)). Silhouette plots show the silhouette coefficient for each sample, which measures how similar a sample is to its own cluster compared to other clusters. Each plot represents a different number of clusters (k). k = 2: the silhouette scores are relatively high, but the plot might indicate that two clusters could be too broad. k = 3: the silhouette scores appear well-distributed with high values, suggesting well-defined clusters. k = 4: the silhouette scores are also relatively high and well-distributed, indicating well-defined clusters. k = 5: the silhouette scores are still good, but there might be a slight decrease compared to k = 3 and k = 4.

The unsupervised k-prototype approach was used to examine the features of each cluster in Table 2. Initially, we applied techniques such as Elbow optimization and Silhouette scores to determine the potential number of clusters. According to the Elbow method, the position where the elbow occurs appears to be around k = 3 or k = 4, as seen in Figure 2. However, the Elbow method is a heuristic and can be subjective, as the “elbow” may not always be well-defined. It is often used in combination with other techniques like Silhouette analysis to determine the optimal number of clusters. The Silhouette coefficient measures how similar an object is to its own cluster compared to other clusters, as depicted in Figure 3. Therefore, considering Figure 2 and Figure 3, the values of k that are most appropriate are k = 3 or k = 4. Both show broad and well-distributed silhouette widths, suggesting well-defined clusters. By combining statistical analysis with practical and clinical considerations, we selected k = 3 (3 clusters).

These clusters created three risk groups: high-risk, medium-risk, and low-risk, based on their incidence of stroke as 9.1%, 6.6%, and 3.2%, respectively. Participants at high risk exhibited numerous distinguishing characteristics, including elevated systolic and diastolic blood pressure, increased non-HDL-c levels, higher fructosamine levels, greater BMI, and higher levels of body fat. Additionally, they had a high prevalence of hypertension and metabolic syndrome. However, their estimated glomerular filtration rate was modest. In contrast, the low-risk categories included younger individuals at the lowest risk for cardiovascular disease.

The supervised model applied five classifier methods, including LR, RF, SVM, XGBoost, and LightGBM. Accuracy, AUC, Recall, Precision, and F1-score are used to evaluate the performance of these models. As demonstrated in Table 3, RF outperformed other models in terms of accuracy, recall, precision, and F1-score. It also had a competitive AUC, making it a strong candidate for the best overall model.

**Figure 4:**
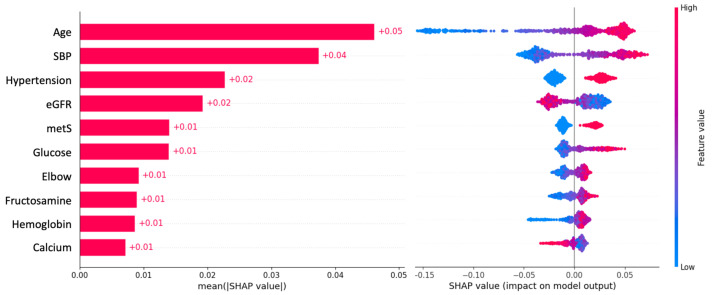
(**left**) shows the contribution levels of these variables to stroke incidence, with the width of the red bar representing their global importance. The SHAP value implies the degree of contribution of a specific feature (variable). The higher the SHAP value is, the larger the model contribution of a specific feature. Figure 4 (**right**), the heat plot of SHAP values reveals the relationships with stroke: red indicates a positive relationship, while blue indicates a negative relationship. Abbreviations: SBP, systolic blood pressure; eGFR, estimated glomerular filtration rate; MetS, metabolic syndrome.

Figure 4 illustrates the additional analysis conducted using SHAP values. RF was utilized to compute the SHAP values, which facilitated the identification of the top most important variables that led to the incidence of stroke.

As shown in Figure 4, age emerged as the most influential predictor among the most important variables. Other significant variables included systolic blood pressure, hypertension, estimated glomerular filtration rate, metabolic syndrome, and blood glucose. Intriguingly, we also discovered that elbow joint thickness, fructosamine level, hemoglobin and serum calcium levels could predict stroke risk. These results are consistent with those reported for the population at high risk, and the most important variables identified by the SHAP method were comparable.

## 4. Discussion

Our study suggested that machine learning techniques can be used for stroke prediction in large-scale population studies. Unsupervised learning, unlike supervised learning, does not rely on pre-labeled outcomes. This makes it particularly valuable for discovering hidden patterns and structures within the data that might not be apparent through traditional methods. Unsupervised k-prototype approaches are appropriate for large datasets that include both categorical and numeric variables, and it aids in elucidating the characteristics of study participants [5,20] and allows us to stratify the population effectively according to their inherent characteristics and risk factors without prior assumptions about the outcomes, enabling targeted interventions and healthcare strategies for each risk category. Moreover, supervised method can also be used in our model, notably the RF, which can be utilized to accurately predict the risk of stroke [21]. Combining unsupervised with supervised machine learning methods provides a comprehensive and consistent approach to identifying risk factors.

Therefore, machine learning techniques can cover a wider variety of variables and identify more complex relationships than traditional methods [7,22,23]. These approaches allow us to stratify individuals based on their risk profiles to confirm the most important factors contributing to stroke risk, thereby enabling more targeted and effective intervention strategies.

### 4.1. Top Most Important Variables and Comparisons with Other Studies

Age, maybe reflecting the duration of risk exposure, was the most significant predictor of non-communicable diseases and stroke. Consistent with prior studies, our investigation also found systolic blood pressure and hypertension [24,25,26,27,28], and estimated glomerular filtration rate (representing chronic kidney disease) [24,29,30] as the most important predictors of stroke incidence.

Participants with a high prevalence of metabolic syndrome exhibited a high incidence of stroke. The metabolic syndrome defines the relationship between diabetes, hypertension, obesity, dyslipidemia, and an increased risk for cardiovascular disease. It is primarily the consequence of an unhealthy diet and a sedentary lifestyle. These modifiable risk factors are becoming more prevalent with the widespread adoption of so-called Western lifestyles [31]. High glucose levels, or hyperglycemia, can damage blood vessels and increase the risk of stroke. Similar to Carson et al. [32], we discovered a relationship between blood glucose level and stroke incidence.

Fructosamine, which encompasses total glycated serum proteins, has gained attention as an alternative glycemic status indicator. It has been acknowledged that it can provide additional insights beyond HbA1c or function as a reliable metric when HbA1c is unreliable. In addition, fructosamine assesses glycemic exposure over a shorter period of time than HbA1c, which examines exposure over the preceding three-month period. This temporal characteristic is advantageous for the monitoring of rapid metabolic fluctuations and adjustments in diabetes therapy [33]. Fructosamine levels have been identified as a potential risk factor for risk of stroke, a finding consistent with previous studies [34,35].

High hemoglobin concentration is also associated with stroke, according to our SHAP research. It is unclear how hemoglobin and serum calcium concentration affect stroke incidence. Our findings imply that stroke incidence is related to hemoglobin concentration, contrary to earlier research [36,37].

Moreover, a majority of studies [38,39] have found an inverse relationship between serum calcium levels and the incidence of stroke. Intriguingly, serum calcium’s potential function as a clinical prognosticator extends beyond ischemic stroke. Hypocalcemia is consistently associated with more severe illness and a higher mortality rate compared to normal calcium levels [40,41], as shown by research on a variety of medical conditions, particularly among critically ill individuals. These results correspond to our own research findings.

In addition, one of our most recent and intriguing discoveries involves the association between elbow joint thickness and the incidence of stroke.

### 4.2. Comparing Our Important Variables and the Variables Used in Framingham and Suita Scores

The Framingham and Suita scores primarily focused on predicting coronary heart disease incidence, but this study focused on stroke incidence. However, coronary heart disease and stroke shared some common risk factors. Some discrepancies were found when comparing our results to the Framingham and Suita scores. The Framingham risk score comprises six coronary risk factors: age, sex, smoking habits, blood pressure, total cholesterol, and HDL cholesterol [42]. The Suita score, developed for the Japanese population, was more accurate in predicting coronary heart disease than the original Framingham risk scores. The Suita score includes similar factors to the Framingham score but also includes an assessment of the CKD stage [43,44].

Our investigation discovered several important predictors of stroke incidence that were not included in either the Framingham or Suita scores, such as elbow joint thickness, fructosamine level, hemoglobin concentration, and calcium level. Nevertheless, age, hypertension, and blood sugar were also revealed as important predictors in our study and the Framingham and Suita scores. We also confirmed the estimated glomerular filtration rate representative for chronic kidney disease, an important variable included in the Suita score.

Hence, our study identified several significant predictors of stroke risk that are similar to findings from other studies. However, some discrepancies were found when comparing our results to the Framingham and Suita scores. The outcomes of this study can assist healthcare professionals in identifying persons at high risk for stroke and implementing preventive measures.

### 4.3. Strengths and Limitations

Our study illustrates that machine learning has capabilities beyond patient categorization. It offers a comprehensive understanding of the specific factors that increase the risk of stroke and provides a realistic plan for applying focused clinical interventions. This stratification of risk groups and identification of the most important risk factors for stroke may improve the accuracy and clinical relevance of stroke preventive efforts, benefiting both individual patient care and population-level health interventions.

But, so far, there are constraints to consider. Our study included only participants from a single region, which may limit the applicability of the results to other populations. Moreover, while machine learning approaches can find more complicated associations between variables, they may be more susceptible to overfitting or developing models that do not generalize well to new data.

It would be advantageous to compare the results of our investigation with those of other studies that have employed machine learning techniques to predict the risk of stroke. This comparison would assist in determining whether or not our findings are compatible with other studies in the field. In addition, it would be advantageous to undertake additional research to validate the findings of our study and assess the efficacy of machine learning algorithms in clinical practice.

## 5. Conclusions

This study found that both unsupervised and supervised learning can effectively develop a stroke prediction model using many predictors from a population-based study. By considering multiple predictors, our research provided a preventive perspective on stroke, facilitating risk assessment, biomarker identification, and identifying novel markers for stroke.

## Figures and Tables

**Table 1:** Characteristics of study participants with and without stroke incidence (healthy Japanese, aged 30–84, Suita study at baseline).

	Overall	Stroke Incidence		*p*-Value
		No	Yes	
	(n = 7389)	(n = 6951, 94.1%)	(n = 438, 5.9%)	
Age, Years	56 [44, 65]	55 [44, 65]	66 [58, 72]	<0.0001
male	3377 (45.7%)	3143 (45.2%)	234 (53.4%)	<0.0001
BMI, kg/m^2^	22.5 (3.01)	22.5 (3.00)	23.1 (3.21)	<0.0001
SBP, mmHg	124 [110, 138]	123 [110, 137]	137 [122, 153]	<0.0001
DBP, mmHg	77.7 (12.2)	77.4 (12.0)	81.2 (13.4)	<0.0001
Smoking, n (%)				0.004
Current	2140 (29.5)	1999 (29.2)	141 (33.3)	
Past	1162 (16.0)	1075 (15.7)	87 (20.5)	
Never	3963 (54.5)	3767 (55.1)	196 (46.2)	
Glucose, mg/dL	95.0 [90.0, 102.0]	95.0 [89.0, 101.0]	99.0 [92.0, 107.0]	<0.0001
Fructosamine, μmol/L	253 (22.3)	253 (22.2)	258 (23.7)	<0.001
Elbow, mm	6.3 (0.6)	6.3 (0.6)	6.4 (0.5)	0.008
Calcium, mg/dL	9.4 (0.4)	9.4 (0.4)	9.3 (0.4)	0.039
Hemoglobin, g/dL	13.9 (1.5)	13.9 (1.5)	14.1 (1.4)	0.001
TG, mg/dL	99.0 [71.0, 144.0]	98.0 [70.0, 143.0]	112.5 [82.0, 163.8]	<0.0001
non-HDL-c, mg/dL	152.6 (36.9)	152.2 (36.9)	158.9 (36.8)	0.0002
eGFR, mL/min/1.73 m^2^	90.0 [73.7, 104.6]	90.3 [74.4, 104.8]	80.0 [66.6, 95.0]	<0.0001
Hypertension, n (%)	2295 (31.1)	2054 (29.5)	241 (55.0)	<0.0001
Diabetes, n (%)	898 (12.2)	798 (11.5)	100 (22.8)	<0.0001
MetS, n (%)	1811 (24.5)	1630 (23.4)	181 (41.3)	<0.0001

Abbreviations: BMI, body mass index; SBP, systolic blood pressure; DBP, diastolic blood pressure; TG, triglycerides; non-HDL-c, non-high-density lipoprotein cholesterol; eGFR, estimated glomerular filtration rate; MetS, metabolic syndrome.

**Table 2:** The characteristic of study participants across the clusters based on unsupervised learning.

	Overall	Stroke Risk			*p*-Value
		High	Medium	Low	
	(n = 7389)	(n = 1974)	(n = 2565)	(n = 2850)	
Stroke incidence, n (%)	438 (5.9)	179 (9.1)	169 (6.6)	90 (3.2)	<0.001
Age, Years	56 [44, 65]	63 [55, 71]	55 [44, 63]	50 [40, 62]	<0.001
Gender					<0.001
Male, n (%)	3377 (45.7)	211 (10.7)	2497 (97.3)	669 (23.5)	
Female, n (%)	4012 (54.3)	1763 (89.3)	68 (2.7)	2181 (76.5)	
BMI, kg/m^2^	22.5 (3.0)	24.0 (2.7)	23.8 (2.6)	20.3 (2.1)	<0.001
Body fat, %	23.2 (6.0)	28.6 (5.6)	20.6 (4.1)	21.8 (5.3)	<0.001
SBP, mmHg	126.3 (20.8)	138.7 (20.0)	129.0 (19.1)	115.4 (16.8)	<0.001
DBP, mmHg	77.6 (11.8)	82.2 (10.8)	81.3 (11.4)	71.1 (9.7)	<0.001
Smoking, n (%)					<0.001
Current	2140 (29.0)	194 (9.8)	1300 (50.7)	646 (22.7)	
Past	1162 (15.7)	157 (8.0)	746 (29.1)	259 (9.1)	
Never	4087 (55.3)	1623 (82.2)	519 (20.2)	1945 (68.2)	
eGFR, mL/min/1.73 m^2^	90.8 (23.7)	86.9 (23.9)	89.1 (22.0)	94.9 (24.4)	<0.001
Hemoglobin, g/dL	13.9 (1.5)	13.3 (1.1)	15.3 (1.0)	13.1 (1.3)	<0.001
TG, mg/dL	99 [71, 144]	116 [87, 159.8]	129 [91, 186]	73 [57, 95]	<0.001
non-HDL-c, mg/dL	152.4 (36.1)	172.2 (34.2)	155.2 (34.2)	136.2 (31.3)	<0.001
HDL-c, mg/dL	54.6 (14.0)	53.3 (13.1)	48.8 (12.5)	60.7 (13.3)	<0.001
Glucose, mg/dL	95 [90, 101]	97 [92, 104]	98 [92.9, 105]	91 [87, 96]	<0.001
Fructosamine, μmol/L	253.2 (22.3)	258.3 (23.0)	251.9 (23.3)	250.8 (20.4)	<0.001
Elbow, mm	6.3 (0.6)	6.1 (0.5)	6.8 (0.4)	6.0 (0.5)	<0.001
Calcium, mg/dL	9.3 (0.4)	9.5 (0.4)	9.4 (0.4)	9.2 (0.4)	<0.001
Hypertension, n (%)	2295 (31.1)	1063 (53.9)	920 (35.9)	312 (10.9)	<0.001
Diabetes, n (%)	898 (12.2)	334 (16.9)	455 (17.7)	109 (3.8)	<0.001
MetS, n (%)	1811 (24.5)	762 (38.6)	997 (38.9)	52 (1.8)	<0.001

Abbreviations: BMI, body mass index; SBP, systolic blood pressure; DBP, diastolic blood pressure; TG, triglycerides; non-HDL-c, non-high-density lipoprotein cholesterol; eGFR, estimated glomerular filtration rate; MetS, metabolic syndrome.

**Table 3:** Performance of different supervised machine learning approaches.

	Accuracy	AUC	Recall	Precision	F1-Score
LR	0.64 ± 0.04	0.68 ± 0.06	0.64 ± 0.04	0.64 ± 0.05	0.64 ± 0.04
RF	0.70 ± 0.05	0.71 ± 0.06	0.70 ± 0.05	0.70 ± 0.06	0.70 ± 0.05
SVM	0.68 ± 0.05	0.73 ± 0.06	0.68 ± 0.05	0.68 ± 0.06	0.68 ± 0.05
XGBoost	0.68 ± 0.05	0.71 ± 0.06	0.68 ± 0.05	0.68 ± 0.05	0.68 ± 0.05
LightGBM	0.66 ± 0.05	0.70 ± 0.06	0.66 ± 0.05	0.67 ± 0.05	0.66 ± 0.05

Abbreviation: AUC, Area Under the Curve; LR, Logistic Regression; RF, Random Forest; SVM, Support Vector Machine; XGBoost, Extreme Gradient Boost; LightGBM, Light Gradient Boosted Machine.

## Data Availability

The dataset examined in this study is not available to the public due to the inclusion of individuals’ personal information.

## Abbreviations

The following abbreviations are used in this manuscript:

SHAP	Shapley Additive Explanations
AUC	Area Under the Curve
VIF	Variance inflation factor
LR	Logistic Regression
SVM	Support Vector Machine
RF	Random Forest
XGBoost	Extreme Gradient Boosting
LightGBM	Light Gradient-Boosting Machine
BMI	Body mass index
SBP	Systolic blood pressure
DBP	Diastolic blood pressure
HDL-c	High-density lipoprotein cholesterol
eGFR	Estimated glomerular filtration rate
MetS	Metabolic syndrome

## Appendix A

**Table A1:** Interpretation for model performance metrics.

Performance Metrics	Definition	Formula	Interpretation
Accuracy	Accuracy is a measure of how many of the total predictions made by the model are correct	$\mathrm{ACC}=\frac{\mathrm{TP}+\mathrm{TN}}{\mathrm{TP}+\mathrm{TN}+\mathrm{FP}+\mathrm{FN}}$	Accuracy tells us the overall correctness of predictions. However, a highly imbalanced dataset can lead to misleadingly high accuracy if the model predicts the majority class most of the time.
AUC	Area under a receiver operating characteristic (AUC-ROC) measures the ability of a model to distinguish between the positive and negative classes by varying the classification threshold		The ROC curve plots the True Positive Rate (Recall) against the False Positive Rate at various threshold values, and AUC-ROC calculates the area under this curve.
Recall	Recall (or Sensitivity) measures the ability of the model to correctly identify positive instances out of all actual positive instances	$\mathrm{Recall}=\frac{\mathrm{TP}}{\mathrm{TP}+\mathrm{FN}}$	Recall quantifies the model’s ability to avoid missing positive cases. It is crucial in scenarios where false negatives (missing actual positive cases) are costly or problematic.
Precision	Precision (or Positive Predictive Value) measures the accuracy of positive predictions made by the model	$\mathrm{Precision}=\frac{\mathrm{TP}}{\mathrm{TP}+\mathrm{FP}}$	Precision focuses on the accuracy of positive predictions.
F1-score	The F1-score is the harmonic mean of precision and recall. It provides a single metric that balances both precision and recall.	$F1\text{-}\mathrm{score}=\frac{2\cdot (\mathrm{Precision}\cdot \mathrm{Recall})}{\mathrm{Precision}+\mathrm{Recall}}$	The F1-score combines the strengths of precision and recall into a single metric.

Abbreviation: AUC, Area Under the Curve; TP, True Positive; TN, True Negative; FP, False Positive; FN, False Negative.
